# Impact of nitric oxide via cardiopulmonary bypass on pediatric heart surgery: a meta-analysis of randomized controlled trials

**DOI:** 10.1186/s13019-024-02953-y

**Published:** 2024-07-19

**Authors:** Minli Zhao, Qiuping Zhang, Yuan Lin, Yukun Chen, Hua Cao

**Affiliations:** 1 Fujian Children’s Hospital (Fujian Branch of Shanghai Children’s Medical Center), College of Clinical Medicine for Obstetrics & Gynecology and Pediatrics, Fujian Medical University, Fuzhou, 350014 China; 2 Fujian Maternity and Child Health Hospital, College of Clinical Medicine for Obstetrics and Gynecology and Pediatrics, Fujian Medical University, Fuzhou, 350000 China; 3 NHC Key Laboratory of Technical Evaluation of Fertility Regulation for Non-Human Primate (Fujian Maternity and Child Health Hospital), Fuzhou, 350000 China

**Keywords:** Nitric oxide, Heart surgery, Cardiopulmonary bypass, Children

## Abstract

**Objective:**

The impact of nitric oxide (NO) administered via cardiopulmonary bypass (CPB) on pediatric heart surgery remains controversial. The objective of this study is to conduct a comprehensive systematic review and meta-analysis to examine the impact of NO administered via CPB on pediatric heart surgery.

**Methods:**

This study searched 7 electronic databases to identify Randomized Controlled Trials (RCTs) on the impact of NO administration during CPB on postoperative outcomes in pediatric heart surgery. The searched databases included Embase, Medline (though PubMed), Cochrane Library, Web of Science, Wan Fang database, China National Knowledge Infrastructure (CNKI), and ClinicalTrials.gov from their inception to November 2, 2022. The included RCTs compared NO administration during CPB with standard CPB procedures or placebo gas treatment in pediatric heart surgery. fixed-effects models and/or random-effects models were used to estimate the effect size with 95% confidence interval (CI). Heterogeneity among studies was indicated by p-values and I^2^. All analyses were performed using Review Manager software (version 5.4) in this study.

**Results:**

A total of 6 RCTs including 1,739 children were identified in this study. The primary outcome was duration of postoperative mechanical ventilation, with the length of hospital and intensive care unit (ICU) stay as the second outcomes. Through a pooled analysis, we found that exogenous NO administered via CPB for pediatric heart surgery could not shorten the duration of postoperative mechanical ventilation when compared with the control group (standardized mean difference (SMD) -0.07, CI [–0.16, 0.02], I^2^ = 45%, *P* = 0.15). Additionally, there were also no difference between the two groups in terms of length of hospital stay (mean difference (MD) -0.29, CI [–1.03, 0.46], I^2^ = 32%, *P* = 0.45) and length of ICU stay (MD -0.22, CI [–0.49 to 0.05], I^2^ = 72%, *P* = 0.10).

**Conclusions:**

This meta-analysis showed that exogenous NO administration via CBP had no benefits on the duration of mechanical ventilation, the length of postoperative hospital, and ICU stay after pediatric heart surgery.

**Supplementary Information:**

The online version contains supplementary material available at 10.1186/s13019-024-02953-y.

## Introduction

Congenital heart disease (CHD) is one of the most common congenital birth defects, affecting approximately 1per100 live births [1]. Each year, around 4,300 newborns in the United States with CHD require surgery in the first few days of their life [2]. Most heart surgeries require cardiopulmonary bypass (CPB), which can probably triggers endothelial barrier function, inflammatory response, and activation of coagulation system [3]. Consequently, 40% of these surgeries can result in myocardial necrosis and ultimately lead to low cardiac output syndrome [4]. CPB is also associated with damage to the central nervous system, lungs, kidneys, gastrointestinal system [5]. Therefore, children are at higher risk for multisystem organ dysfunction after undergoing congenital heart surgery [6, 7].

NO is an important vasoactive molecule in the body. Studies have shown that it can relax pericytes and vascular smooth muscle cells by binding to the cytosolic guanylate cyclase, leading to a decrease in intracellular Ca^2+^ [8]. NO helps maintain cardiovascular system homeostasis through vascular dilatation, anti-aggregation and anti-adhesion effects [9]. It has also been demonstrated to protect the myocardium by alleviating ischemia-reperfusion injury duringCPB [10]. However, CPB can increase level of free hemoglobin, which removes substantial amounts of NO from the body [11]. Deficiency in endogenous NO may result in organ damage, suggesting that supplementation with exogenous NO during CPB could reduce the incidence of morbidity and mortality after heart surgery [12, 13]. Several studies have reported the benefits of delivering exogenous NO directly into the CPB for adult undergoing heart surgery [14, 15]. Additionally, Checchia et al. found that infants undergoing congenital heart surgery who were treated with NO showed improvement in important clinical outcomes such as postoperative duration mechanical ventilation and length of ICU stay [16]. In the contrast, a recent clinical study reported that NO delivery via CPB circuits had no significant impact on postoperative recovery, leading them to discourage the use of NO delivery into an CPB oxygenator during pediatric heart surgery [17].

Overall, we believe that a pooled analysis of the effects of NO supplementation via CPB on the postoperative recovery of infants undergoing heart surgery is of great clinical significance. This study aims to perform a systematic review and pooled analysis on the latest updated data to assess the impact of NO via CPB on pediatric heart surgery.

## Methods

### Guidance and protocol

This meta-analysis was conducted based on the Preferred Reporting Items for Systematic Reviews and Meta-analysis (PRISMA) statement [18]. Protocol of this study has been registered on the PROSPERO (Registration number: CRD42022376413) on November 27, 2022. This study was conducted in accordance with the Declaration of Helsinki and received approval from the local institutional review board.

### Search strategy

A systematic and comprehensive search was conducted using electronic databases, including Embase, Medline (through PubMed), the Cochrane library, Web of Science, Wan Fang database, and the China National Knowledge Infrastructure (CNKI), to select relevant literature from inception to November2, 2022. Clinical trials were identified by searching ClinicalTrials.gov. The search used a combination of free terms and Mesh terms, including “Nitric Oxide” and “Cardiopulmonary Bypass” and “heart surgery”. No language restrictions were applied. Two independent authors conducted the literature search and screening. Any disagreements between authors were resolved through consultation with a third author until a consensus was reached.

### Inclusion criteria and exclusion criteria

#### Inclusion criteria

RCTs with a published full-text article were included if they met the following criteria: (1) RCTs investigating the impact of NO administration during CPB on postoperative outcomes in pediatric heart surgery; (2) Setting of CPB for heart surgery in children (< 14 years old); (3) Patients receiving only NO during CPB compared to those receiving placebo or standard care without additional treatments; (4) Reporting relevant outcomes such as postoperative duration of mechanical ventilation, length of hospital stay, and length of ICU stay.

#### Exclusion criteria

Studies were excluded if they: (1) Involved inhalation NO before or after CPB; (2) Multiple duplicate publications of the same data; (3) Were animal experiment.

### Data extraction

Qiuping Zhang and Yuan Lin independently conducted data extraction thoroughly reviewing the full text. Any disagreements were resolved by through consultation with a third investigator. The extracted data included the author’s name, publication year, country, the population size, sex, comparator, the protocol of NO administration, the setting for NO Initiation, and key postoperative outcome.

### Risk of bias assessment

Two authors independently assessed the risk of bias in each study using the Cochrane Collaboration’s Tool [19]. The risk of bias based on selection bias, performance bias, detection bias, attrition bias, reporting bias, and other bias. If a study did not explicitly state a specific bias, despite efforts to contact the authors for clarification, the item was classified as having an “unclear” risk.

### Statistical analysis

Review Manager 5.4 was utilized for all the statistical analysis [20]. Given that indicators in this study were all continuous variables with varying units, we employed the mean difference (MD) / standardized mean difference (SMD) for analyzing outcomes, providing a 95% confidence interval (CI). Standard deviation (SD) were calculated using sample size and mean values in cases where SD was not provided. Heterogeneity was assessed using I^2^ statistics. For analyses without significant heterogeneity (I^2^ < 50%), a fixed-effects model was utilized; for those with significant heterogeneity(I^2^ ≥ 50%), a random-effects model was applied [21]. A *P*-value < 0.05 was considered statistically significant.

## Results

### Literature characteristics

**Fig. 1:**
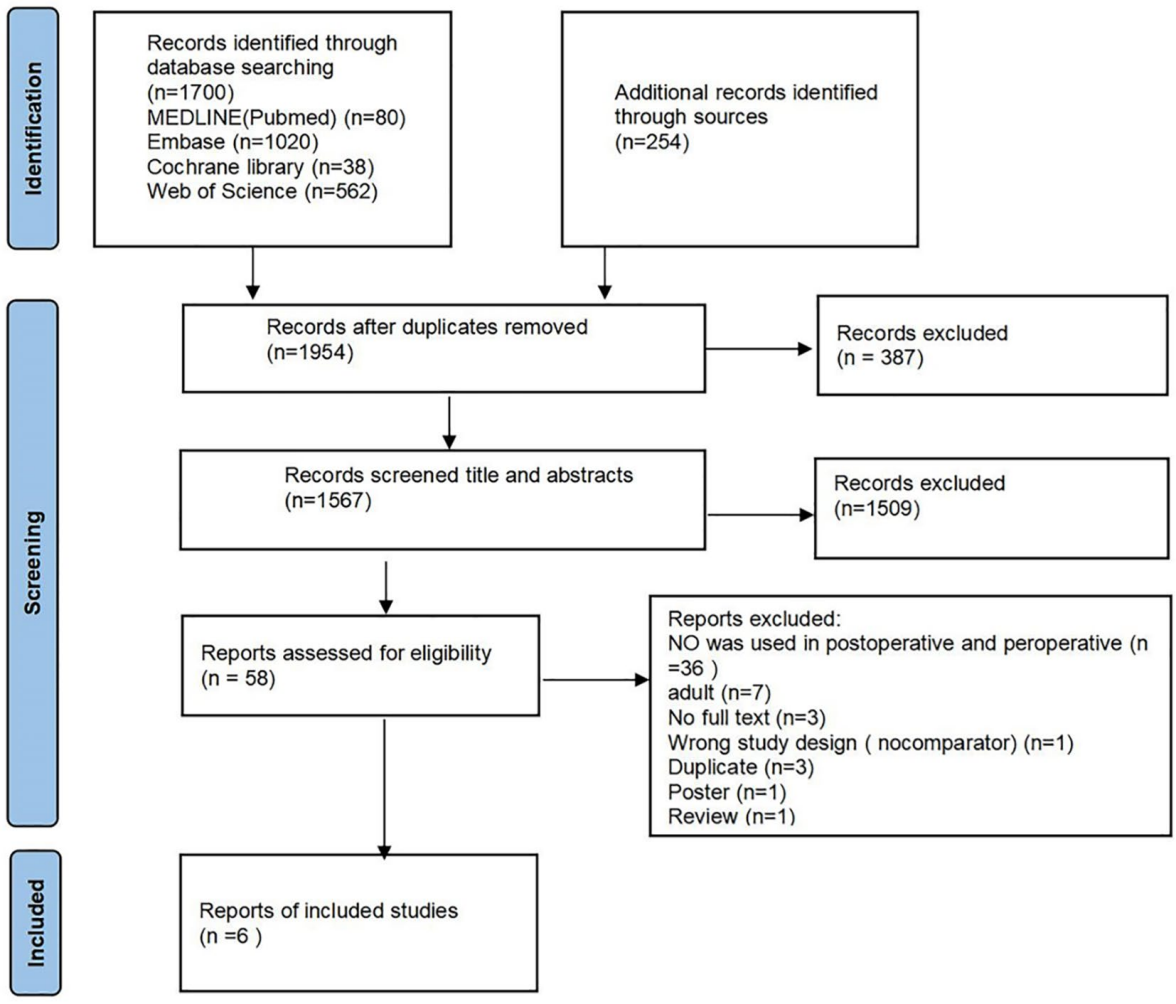
Flowchart of selection of studies included in the meta-analysis

According to the search strategy, a total of 1971 potentially relevant studies were identified from the electronic database. After eliminating duplicates, irrelevant references, and unqualified references, six studies involving 1739 children were included in the final analysis (Fig. 1).

The baseline characteristic of all included studies are summarized in Table 1. All studies were prospective and randomized in design, conducted between 2013 and 2022. The patients in our study were children whose age under 14 years old. No statistically significant differences were found in the baseline characteristics between the experimental and the control groups. Postoperative mechanical ventilation duration after CPB was reported in five studies, hospital stay after CPB was included in six studies, and ICU stay length after CPB in four studies.

**Table 1:** Baseline characteristic of all the included studies

Author	Year	Country	*N*	Female (%)	Age (mean ± SD)	Comparator	The protocol of NO therapy	NO initiation Setting
Schlapbach et al.	2022	Australia	NO:679 Control:685	NO:266 Control:317	total:21.2 ± 23.5 weeks	standard care	20ppm, through CPB	Operating room
Kolcz et al.	2022	Poland	NO:48 Control:49	NO:26 Control:28	NO:897.4 ± 242.4 days Control:964.6 ± 228.7 days	standard care	20ppm, through CPB	Operating room
Niebler et al.	2021	USA	NO:18 Control:22	unclear	NO:100.6 ± 77.7 days Control:112.4 ± 92.5 days	placebo	20ppm, through CPB	Operating room
Elzein et al.	2020	USA	NO:12 Control:12	NO:4 Control:5	NO:5.67 ± 1.87 days Control:5.92 ± 1.78 days	placebo	40ppm, through CPB	Operating room
James et al.	2016	Australia	NO:101 Control:97	NO:40 Control:42	NO:6.0(1.0–43.0) month Control:4.0(1.0–38.0) month	standard care	20ppm, through CPB	Operating room
Checchia et al.	2013	USA	NO:8 Control:8	NO:1 Control:4	NO:191 ± 112 days Control:216 ± 114 days	placebo	20ppm, through CPB	Operating room

*Abbreviation* NO: nitric oxide; CPB: cardiopulmonary bypass; SD: standard deviation

### Bias assessment

**Fig. 2:**
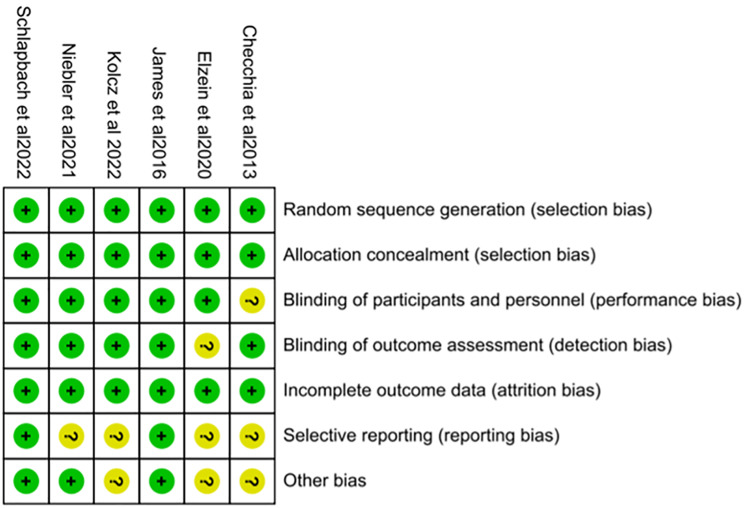
Risk of bias summary table; *Abbreviation* Yellow (?): unclear risk of bias; Green (+): low risk of bias

All included studies were randomized controlled trials. The Cochrane risk of bias analysis for these studies is presented in Fig. 2, showing an insignificant risk of bias across all studies included in this meta-analysis.

### Nitric oxide

In all studies, NO was administered during CPB in the operating room. However, NO was not applied into the CPB oxygenator in the standard care or placebo group.

### Primary outcome and other outcomes

#### Duration of mechanical ventilation

**Fig. 3:**
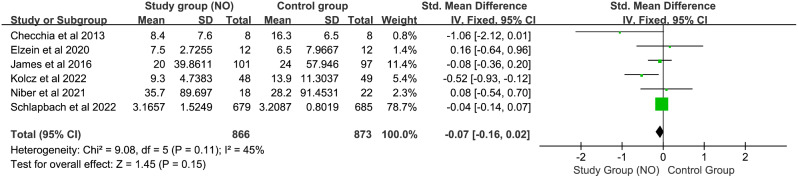
Forest plot for duration of mechanical ventilation mean difference between control group and nitric oxide group. *Abbreviation* IV: inverse variance; CI: confidence interval; Std. Mean Difference: Standard Mean Difference

As depicted in Fig. 3, six studies including 1739 children (873 in the control group and 866 in the NO group) were included in the analysis of postoperative mechanical ventilation duration. The Q-statistic yielded a p-value of 0.15 and the I^2^-statistic was 45%, indicating no significant heterogeneity among studies. Therefore, a fixed-effects model was applied to pooled analysis. Ultimately, our analysis suggests that NO does not reduce the duration of postoperative mechanical ventilation (SMD − 0.07, CI [–0.16, 0.02], I^2^ = 45%, z = 1.45, *P* = 0.15).

#### Length of Hospital stay

**Fig. 4:**
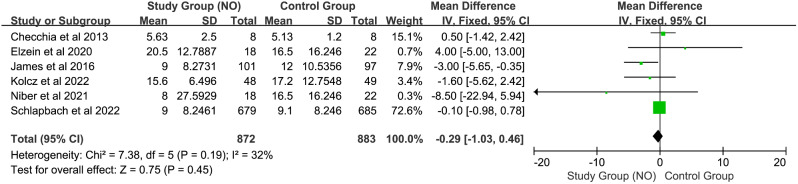
Forest plot for length of hospital stay mean difference between control group and nitric oxide group. *Abbreviation* IV: inverse variance; CI: confidence interval

As illustrated in Fig. 4, six studies including 1739 children (873 in the control group and 866 in the NO group) investigated the length of hospital stay. Given an I^2^ < 50%, a fixed-effects model was employed. The pooled analysis indicates that the use of NO in SPB did not significantly affect the length of hospital stay (MD -0.29, CI [–1.03, 0.46], I^2^ = 32%, z = 0.76, *P* = 0.45).

#### Length of ICU stay

**Fig. 5:**
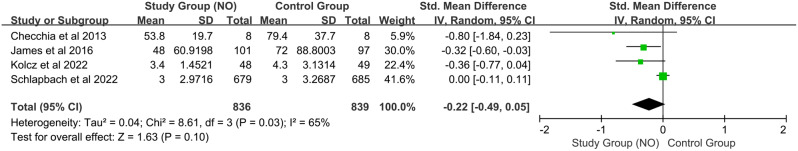
Forest plot for length of intensive care unit stay mean difference between control group and nitric oxide group. Abbreviation IV: inverse variance; CI: confidence interval; Std. Mean Difference: Standard Mean Difference

As shown in Fig. 5, four studies including 1675 children (839 in the control group and 836 in the NO group) investigated postoperative length of ICU stay. The I^2^-statistic showed a significant heterogeneity of 65%, leading us to use a random-effects model. However, due to the limited number of studies included, Egger’s regression could not be conducted to assess publication bias. The analysis revealed no significant difference in the postoperative length of ICU stay between two groups (SMD − 0.22, CI [–0.49, 0.05], I^2^ = 65%, z = 1.63, *P* = 0.10).

## Discussion

The impact of inhaled NO during heart surgery for CHD on perioperative outcomes has been widely investigated. However, the clinical significance of NO administration via CPB in congenital pediatric heart surgery remains controversial. This meta-analysis includes six studies with a total of 1739 children, primarily infants under one year of age. The primary findings revealed that NO administered via CPB had no benefits on the duration of mechanical ventilation, length of postoperative hospital stay and ICU stay.

Salvatore et al. found, through pooled analysis, that perioperative inhaled NO has little effect on patients with pulmonary hypertension undergoing heart surgery [22]. Their review was very comprehensive, but it did not emphasize the impact of delivering NO through CPB on heart surgery. Joseph Mc Loughlin et al. conducted the first systematic review on **t**he impact of exogenous NO via CPB on patients undergoing heart surgery [23]. Despite including both adults and children in their analysis, only three studies were eligible, each with a small sample size. Their study also indicated no significant differences in the duration of mechanical ventilation between control group and NO group after heart surgery. Consistent with Joseph’s results, we found that NO had no benefits on the duration of mechanical ventilation, length of postoperative hospital stay and ICU stay after heart surgery, despite differences in the number of included articles and study populations compared to their studies.

NO synthesized by the vascular endothelium can inhibit neutrophil-endothelial adhesion and platelet activation. The mechanism of NO in treating pulmonary hypertension in neonates is well established [24]. Recently, NO has been found to play other significant roles in clinical practice, such as suppressing the systemic inflammatory response (SIR) and reducing ischemia-reperfusion injury of vital organs [25, 26]. It has been reported that CPB can trigger the release of a series of proinflammatory cytokines and Lymphocyte activation [27]. Anesthetic strategies and steroid administration are common methods to mitigate CPB-induced trauma [28]. Compared with the above methods, adding NO to CPB is a safe and convenient strategy. Consequently, increasing numbers of clinical studies more and more clinical studies are investigating the potential benefits of adding NO to CPB for postoperative recovery.

In the past decade, numerous studies had reported that adding NO to the CPB circle can reduce heart and lung injuries [29, 30]. Additionally, it is well known that inhaled NO can alleviate pulmonary arterial hypertension during cardiac surgery in infants [31–33]. However, RCTs on the effects of adding NO to the CPB circuit during heart surgery for children are rare. The first RCT reported that NO administered via CPB during infant heart surgery significantly shortened length of ICU stay and the duration of postoperative mechanical ventilation [16]. Subsequent studies have investigated the application of NO via CPB in cardiac surgery for younger children. Schlapbach et al. found that NO administered via CPB had little or no influence on the number of ventilator-free within 30 days and other outcomes [17]. Kolcz et al. found that NO had a significant effect on inflammatory factors and indicators of lung and myocardial injury [25]. Niebler et al. concluded that NO had no impact on number or activation of platelet in infants undergoing heart surgery and found no differences in adverse events between the study and control groups [34]. James et al. focused on the influence of NO via CPB on low cardiac output syndrome [35]. Checchia et al. found that adding NO to the CPB could protect myocardial and reduced length of ICU stay. The small sample sizes may have contributed to a larger confidence interval in the pooled analysis of postoperative length of ICU stay [16]. Elzein et al. observed that NO had no impact on postoperative recovery despite its ability to protect the myocardium [2]. This meta-analysis provided a comprehensive analysis of studies that had common indicators and were controversial. Mechanical ventilation was the most effective way to provide respiratory support for infants undergoing heart surgery. Duration of postoperative mechanical ventilation was an important indicator to reflect the recovery of respiratory function after surgery [36]. However, we found that administration of NO during CPB did not reduce the duration of postoperative mechanical ventilation or improve the prognosis. In addition, NO via CPB also had no benefits on the length of postoperative hospital and ICU stay. The recovery after pediatric heart surgery involved multiple factors, including the type of surgery, individual patient differences, postoperative management strategies, and more [37–39]. The effects of nitric oxide might have been obscured by these complex factors, making it difficult for its impact to be significant on its own, which might explain our results. Certainly, more clinical and basic research was needed to further validate the role of NO in pediatric cardiac surgery, in order to better guide clinical practice.

This meta-analysis had several limitations. Firstly, there is a significant disparity in the number of patients among the enrolled studies, with small sample sizes potentially affecting the reliability of the results. Secondly, for data not normally distributed, results were presented as median and interquartile range. Converting these medians into means and standard deviations may introduce errors. Thirdly, the limited number of included studies in this research prevented subgroup analyses (such as grouping by age or the amount of NO administration) and testing for publication bias, which might impact the reliability of the findings. Therefore, further studies are needed to validate our results.

## Conclusions

This meta-analysis shows that NO has no effect on the duration of mechanical ventilation, hospital stay, or ICU stay after heart surgery with CPB. Given the heterogeneity and limitations of the included studies, along with the variation in sample sizes, the overall quality of the evidence may be suboptimal. Further research is needed to explore the mode and timing of NO in pediatric heart surgery.

### Electronic supplementary material

Below is the link to the electronic supplementary material.

Supplementary Material 1

## Data Availability

No new data were created or analyzed in this study. Data sharing is not applicable to this article.
