# Supplementary material for: Impact of nitric oxide via cardiopulmonary bypass on pediatric heart surgery: a meta-analysis of randomized controlled trials
Source: J Cardiothorac Surg. 2024 Jul 19;19:461. doi: 10.1186/s13019-024-02953-y (PMC11258894; doi:10.1186/s13019-024-02953-y)
Supplement: Supplementary file 1 — Supplementary Material 1 [file 13019_2024_2953_MOESM1_ESM.doc]

**Pubmed检索**

**(((("Nitric Oxide"[Mesh]) OR (((((((((((Oxide, Nitric[Title/Abstract]) OR (Nitrogen Monoxide[Title/Abstract])) OR (Monoxide, Nitrogen[Title/Abstract])) OR (Nitric Oxide, Endothelium-Derived[Title/Abstract])) OR (Endothelium-Derived Nitric Oxide[Title/Abstract])) OR (Nitric Oxide, Endothelium Derived[Title/Abstract])) OR (Endogenous Nitrate Vasodilator[Title/Abstract])) OR (Nitrate Vasodilator, Endogenous[Title/Abstract])) OR (Vasodilator, Endogenous Nitrate[Title/Abstract])) OR (Mononitrogen Monoxide[Title/Abstract])) OR (Monoxide, Mononitrogen[Title/Abstract])))) AND (("Cardiopulmonary Bypass"[Mesh]) OR ((((((((Heart-Lung Bypass[Title/Abstract]) OR (Bypass, Heart-Lung[Title/Abstract])) OR (Bypasses, Heart-Lung[Title/Abstract])) OR (Heart Lung Bypass[Title/Abstract])) OR (Heart-Lung Bypasses[Title/Abstract])) OR (Bypass, Cardiopulmonary[Title/Abstract])) OR (Bypasses, Cardiopulmonary[Title/Abstract])) OR (Cardiopulmonary Bypasses[Title/Abstract])))) AND (("Thoracic Surgery"[Mesh]) OR ((((("Surgery, Thoracic"[Title/Abstract]) OR ("Surgery, Cardiac"[Title/Abstract])) OR ("Surgery, Heart"[Title/Abstract])) OR ("Heart Surgery"[Title/Abstract])) OR ("Cardiac Surgery"[Title/Abstract])))** Sort by: **Most Recent**

****Embase检索****

| #11 | #7 AND #10 | 1020 |
| --- | --- | --- |
| #10 | #8 OR #9 | 71701 |
| #9 | 'heart-lung bypass':ab,ti OR 'bypass, heart-lung':ab,ti OR 'bypasses, heart-lung':ab,ti OR 'heart lung bypass':ab,ti OR 'heart-lung bypasses':ab,ti OR 'bypass, cardiopulmonary':ab,ti OR 'bypasses, cardiopulmonary':ab,ti OR 'cardiopulmonary bypasses':ab,ti | 203 |
| #8 | 'cardiopulmonary bypass'/exp OR 'cardiopulmonary bypass' OR (cardiopulmonary AND ('bypass'/exp OR bypass)) | 71609 |
| #7 | #3 AND #6 | 7457 |
| #6 | #4 OR #5 | 966664 |
| #5 | 'surgery, thoracic':ab,ti OR 'surgery, cardiac':ab,ti OR 'surgery, heart':ab,ti OR 'heart surgery':ab,ti OR 'cardiac surgery':ab,ti | 85913 |
| #4 | 'thoracic surgery'/exp OR 'thoracic surgery' OR (thoracic AND ('surgery'/exp OR surgery)) | 954163 |
| #3 | #1 OR #2 | 289764 |
| #2 | 'oxide, nitric':ab,ti OR 'nitrogen monoxide':ab,ti OR 'monoxide, nitrogen':ab,ti OR 'nitric oxide, endothelium-derived':ab,ti OR 'endothelium-derived nitric oxide':ab,ti OR 'nitric oxide, endothelium derived':ab,ti OR 'endogenous nitrate vasodilator':ab,ti OR 'nitrate vasodilator, endogenous':ab,ti OR 'vasodilator, endogenous nitrate':ab,ti OR 'mononitrogen monoxide':ab,ti OR 'monoxide, mononitrogen':ab,ti | 2570 |
| #1 | 'nitric oxide'/exp OR 'nitric oxide' OR (nitric AND ('oxide'/exp OR oxide)) |  |

**Cochrane library 检索**

**#1 MeSH descriptor: [Nitric Oxide] explode all trees 2261**

**#2 (“Oxide, Nitric”):ti,ab,kw OR (“Nitrogen Monoxide”):ti,ab,kw OR (“Monoxide, Nitrogen”):ti,ab,kw OR (“Nitric Oxide, Endothelium-Derived”):ti,ab,kw OR (“Endothelium-Derived Nitric Oxide”):ti,ab,kw (Word variations have been searched) 118**

**#3 (“Nitric Oxide, Endothelium Derived”):ti,ab,kw OR (“Endogenous Nitrate Vasodilator”):ti,ab,kw OR (“Nitrate Vasodilator, Endogenous”):ti,ab,kw OR (“Vasodilator, Endogenous Nitrate”):ti,ab,kw OR (“Mononitrogen Monoxide”):ti,ab,kw (Word variations have been searched) 2**

**#4 (“Monoxide, Mononitrogen”):ti,ab,kw (Word variations have been searched) 0**

**#5 #1 or #2 or #3 or #4 2356**

**#6 MeSH descriptor: [Cardiopulmonary Bypass] explode all trees 2818**

**#7 (“Heart-Lung Bypass”):ti,ab,kw OR (“Bypass, Heart-Lung”):ti,ab,kw OR (“Bypasses, Heart-Lung”):ti,ab,kw OR (“Heart Lung Bypass”):ti,ab,kw OR (“Heart-Lung Bypasses”):ti,ab,kw (Word variations have been searched) 14**

**#8 (“Bypass, Cardiopulmonary”):ti,ab,kw OR (“Bypasses, Cardiopulmonary”):ti,ab,kw OR (“Cardiopulmonary Bypasses”):ti,ab,kw (Word variations have been searched) 7152**

**#9 #6 or #7 or #8 7163**

**#10 MeSH descriptor: [Thoracic Surgery] explode all trees 180**

**#11 ("Surgery, Thoracic"):ti,ab,kw OR ("Surgery, Cardiac"):ti,ab,kw OR ("Surgery, Heart"):ti,ab,kw OR ("Heart Surgery"):ti,ab,kw OR ("Cardiac Surgery"):ti,ab,kw (Word variations have been searched) 12629**

**#12 #10 or #11 12773**

**#13 #5 and #9 and #12 38**

****Web of science 检索****

1: 1: (((((((((((TS=("Nitric Oxide")) OR TS=("Oxide, Nitric")) OR TS=("Nitrogen Monoxide")) OR

TS=("Monoxide, Nitrogen")) OR TS=("Nitric Oxide, Endothelium-Derived")) OR

TS=("Endothelium-Derived Nitric Oxide")) OR TS=("Nitric Oxide, Endothelium Derived")) OR

TS=("Endogenous Nitrate Vasodilato")) OR TS=("Nitrate Vasodilator, Endogenous")) OR

TS=("Vasodilator, Endogenous Nitrate")) OR TS=("Mononitrogen Monoxide")) OR

TS = ("Monoxide, Mononitrogen")

2: ((((((((TS=("Cardiopulmonary Bypass")) OR TS=("Heart-Lung Bypass")) OR TS=("Bypass,

Heart-Lung")) OR TS=("Bypasses, Heart-Lung")) OR TS=("Heart Lung Bypass")) OR

TS=("Heart-Lung Bypasses")) OR TS=("Bypass, Cardiopulmonary")) OR TS=("Bypasses,

Cardiopulmonary")) O R TS = ("Cardiopulmonary Bypasses")

3. (((((TS=("Thoracic Surgery")) OR TS=("Surgery, Thoracic")) OR TS=("Surgery, Cardiac")) OR

TS = ("Surgery, Heart")) O R TS = ("Heart Surgery")) O R TS = ("Cardiac Surgery")

1. 1 and 2 and 3 562
